# A new explicit numerical scheme for enhancement of heat transfer in Sakiadis flow of micro polar fluid using electric field

**DOI:** 10.1016/j.heliyon.2023.e20868

**Published:** 2023-10-12

**Authors:** Yasir Nawaz, Muhammad Shoaib Arif, Kamaleldin Abodayeh, Muhammad Usman Ashraf, Mehvish Naz

**Affiliations:** a Department of Mathematics, Air University, PAF Complex E-9, Islamabad, 44000, Pakistan; b Department of Mathematics and Sciences, College of Humanities and Sciences, Prince Sultan University, Riyadh, 11586, Saudi Arabia; c Department of Sciences and Humanities, National University of Computer and Emerging Sciences, Islamabad, Pakistan; d Department of Mathematics, Comsats University Islamabad, Wah Campus, G. T. Road, Wah Cantonment, Pakistan

**Keywords:** Predictor-corrector scheme, Stability region, High accuracy, Micro polar fluid, Electric field

## Abstract

This article suggests a fourth-order numerical approach for solving ordinary differential equations (ODEs) that are both linear and nonlinear. The suggested scheme is an explicit predictor-corrector scheme. For linear ODE, the proposed numerical scheme's stability area is discovered. The proposed strategy yields the same stability region as the traditional fourth-order Runge-Kutta method. In addition, partial differential equations (PDEs) are used to develop the mathematical model for the flow of non-Newtonian micro-polar fluid over the sheet and heat and mass transit using electric field effects. These PDEs are further transformed into dimensionless boundary value problems. Boundary value problems are resolved using the proposed shooting-based scheme. The findings show that increasing values of ion kinetic work and Joule heating parameters cause the temperature profile to climb. The results produced by the suggested strategy are compared to those discovered through earlier studies. The results of this study could serve as a starting point for future fluid-flow investigations in a secure industrial environment.

## 1 Introduction

Microstructure fluids are composed of randomly oriented circular particles possessing the character of rigid rotation and halted movement in a viscous medium. Such fluids can also be called micropolar. It is worth mentioning that Navier-Stokes equations have no clarification for micro and nanoscale phenomena. In contrast, microfluid dynamics (MFD) can explain physical phenomena in micro and nano scales due to its freedom of circulation. Non-Newtonian fluids, polymer fluids, liquid crystals, and blood flows contain intrinsic polarities and are physical examples of micropolar fluids. Micro-polar fluid dynamics is a valuable tool to model physical processes like the presence of fumes or particles in gas emitted out of chimneys. Practical uses of heat transfer through the perforated medium are crude oil extraction, groundwater pollution, and geothermal systems.

Eringen [1,2] pioneered the micropolar fluid theory that demonstrates few microscopic effects produced due to the microstructure nature and the micromotion of fluid movement. Ali et al. [3] investigated natural convective laminar boundary layer flow using a flat plate. Harutha and Devasena [4] carry out further investigation. The viscous incompressible micropolar liquid was the subject of their investigation because of the buoyancy forces that let it convection through a porous media and reach a stagnation point over a vertical surface. The study of the two-dimensional parallel shear flow of a linear micropolar fluid was done by Hudimoto and Tokuoka [5].

The major findings of this study were based on the comparative analysis of simple and colloidal suspension. Stable micro-polar free convection fluid flow was reported by Rees and Pop [6] from a flat plate. Elbarbary [7] considered Chebyshev's finite difference method for solving the boundary layer flow equations. Analysis of free convection micropolar fluid flow over an inclined stretching sheet having heat and mass transportation was done by Nandhini and Ramya [8]. Hassanien and Glora [9] did additional work on the micropolar fluid dynamics and formulated a study of micropolar fluid on a non-isothermal stretching sheet using heat transportation; however, micro polar fluid and mass transfer over a nonlinear stretching plate were done by Ahmad et al. [10]. Khonsari and Brewe [11] studied this liquid's parameters using finite-length lubrication. Results depicted a more efficient load-carrying capacity of micro-polar fluids than Newtonian fluids. Ezzat and Abd-Ellal [12] demonstrated that free convection currents with one relaxation time affect the viscoelastic conduction fluid flow in porous media.

The hydromagnetic non-Newtonian fluid flow was explored by Edlabe and Mohammed [13] in terms of heat and mass transfer. In addition to their findings, Edlabe and Ouaf [14] introduced the parameters of ohmic heating and viscous dissipation to the hydromagnetic flow of micropolar fluids. Aydin and Pop [15] investigated the steady, laminar, and two-dimensional natural convection flow with heat transportation in a square container for micropolar fluids. Micro-polar fluid vibratory flow in an annular region with constriction was studied by Muthu et al. [16] by varying the outer tube radius. Uneven convection of a micropolar fluid was presented by Glora [17] by utilizing a vertical plate.

On the other hand, Hsu and Wang [18] performed a numerical investigation of mixed convective micropolar fluid in a square cavity with a localized heat source effect. Micro-polar fluid dynamics on a double infinite vertical straight surface was done by Lok et al. [19]. Furthermore, Zakaria [20] introduced the Laplace transformation with the ϵ-algorithm technique to numerically identify and investigate the electrically conducting micropolar fluid flow with the effects of a transverse magnetic field.

Today, boundary layer control using electromagnetic body forces against a layer forms the primary basis of experimental and numerical research [[21], [22], [23], [24]]. The ionic wind is induced on a flat surface by electro-fluid dynamic actuators such as surface corona electrical discharge, which results in the change of velocity and air profile near the plate. Hence, it delayed the transition of the layer into turbulent form and reduced the total drag [25,26]. Weak conductive fluids like seawater utilized Lorentz forces to control flow separation at hydrofoils [27].

The electric field carried ionic wind current, which became a cause of electric force generation and joule heating. Heat transportation by convection near the boundary was influenced by the electric field's motion induced by electric force and joule heating [28]. Corona discharge considerably surges the local heat transportation coefficients between gas flow and a solid boundary, usually air [[29], [30], [31], [32], [33], [34]]. The electric wind generated by ionized air produced efficient convection and raised heat transportation.

The most recent study [35] used stream-wise electric body forces to describe the simplified isothermal boundary layer control problem. According to Mendes and Dente [36] explored the boundary layer equations using a group-theoretical study for an assumed ion charge density profile that decreases away from the wall. The Williamson micropolar fluid flow has been investigated in Ref. [37]. The governing equations have been reduced into the system of ODEs and solved with the Runge-Kutta method based on the shooting approach. It was seen that the magnetic field and non-Darcy parameters were responsible for the de-escalation of the velocity of the fluid. Time-dependent MHD micropolar fluid was also explored in Ref. [38] over a three-dimensional variable stretching sheet. The reduced ODEs have been solved by the homotopy perturbation method and the Runge-Kutta method based on the shooting approach. It was mentioned that the investigation might have been profitable for the exotic lubricants, artificial fibers, and polymer fluids. In Ref. [39], researchers try to build a model that accurately depicts the flow and thermal characteristics of heat generation (sinking) in an MHD Maxwell nanofluid moving over a stretchy surface. The flow dynamics in highly nonlinear PDEs are typically presented employing Fourier and Fick's laws. The ideal 1-dimensional system of Lie sub-algebras, associated invariants, and similarity transformations is shown in Ref. [40]. These adjustments help us narrow down the number of variables in the flow model under consideration. The controlling partial differential equations are reduced twice to ordinary differential equations, which we successfully implement.

Various analytical and numerical techniques exist in the literature for finding the solutions to ODEs. The numerical methods may consume less time than analytical methods for solving differential equations. However, analytical methods' main advantage is finding an exact solution in some cases. Numerical or computational methods might have been considered for finding approximate solutions to differential equations. The computational methods can be divided into explicit and implicit types categories. Most explicit methods have small stability regions but do not require any iterative method for finding solutions to differential equations. The proposed method in this contribution is an explicit technique formulated on two grid points and is conditionally stable. The proposed technique requires the evaluation of the first derivative as well as the second-order derivatives of the dependent variable. In contrast, most existing methods only require the information of the first-order derivative of the dependent variable of the given differential equation.

A mathematical model for the flow of micropolar fluid over the moving sheet under the influence of the electric field, thermal radiation, and chemical reaction is built in addition to the suggested numerical method. The boundary conditions are stated at the plate and the distance from the plate. The abrupt shift of the plate in this situation causes the fluid to flow. A set of ordinary differential equations are created from the governing equations. Additionally, the suggested scheme is used to solve these equations. Finding another derivative of the differential equation is the suggested scheme's drawback. However, it provides fourth-order accuracy in two stages, whereas the existing Runge-Kutta method provides fourth-order accuracy in four stages. The proposed scheme provides the same stability region as the traditional fourth-order Runge-Kutta method. The suggested scheme solves first-order ordinary differential equations that arose in the flow phenomena. The shooting method is based on the Matlab solver fsolve for solving equations. The generated set of differential equations is also resolved using the existing Euler method and the Matlab solver bvp4c. The technique has another weakness in solving boundary value problems besides the one already stated in this article. Another approach, called the shooting method, can be employed to overcome this drawback.

Fluids directly contribute to the production of power or electricity in hydroelectric power plants, aeroplanes, and automobiles. Fluids generate electricity indirectly in thermal and nuclear power plants, although they comprise most of these applications. These are the applications of fluid mechanics. The paper is organized as follows:

A numerical scheme is first offered in section 2 using Taylor series expansions. In section 3, the stability of the numerical scheme for linear equations is given, and the consistency of the proposed scheme is also shown. Finally, the mathematical model for the flow over the sheet is constructed, and the findings are further examined in sections 4, 5. The result, discussion, and conclusion are shown in sections 6, 7. Below, we'll go over the situation's most crucial details.

i. The fourth-order numerical approach has been proposed as a two-step method for solving first-order linear and nonlinear ordinary differential equations (ODEs).
ii. The proposed mathematical model of the flow of a non-Newtonian micro-polar fluid over the sheet, including the transfer of heat and mass due to electric field effects, is solved by developing a computational numerical system.
iii. The suggested numerical approach achieves high precision and the projected order of convergence.
iv. Prove that the proposed scheme is reliable and consistent.
v. The scheme's effectiveness can be demonstrated by applying it to a few nonlinear situations and problems in the real world.

## 2 Proposed numerical scheme

A general sort of first-order differential equation is selected to begin the construction process of the suggested numerical system. Consider the differential equation as evidence for this,

(1) $$y^{\prime }=f\left(y\right)$$

subject to the initial condition

$$y\left(0\right)=\alpha_{1}$$

where $\alpha_{1}$ is constant.

The presented numerical scheme is a predictor-corrector or two-stage numerical scheme. The first/predictor stage of the numerical scheme is given as:

(2) $${\bar{y}}_{i}=y_{i-1}+hy_{i-1}^{\prime }+h^{2}y_{i-1}^{\prime \prime }$$

where h is the step size.

The second/corrector stage of the proposed numerical scheme is expressed as:

(3) $$y_{i}=y_{i-1}+h\left(a{\bar{y}}_{i}^{\prime }+by_{i-1}^{\prime }\right)+h^{2}\left(c{\bar{y}}_{i}^{\prime \prime }+dy_{i-1}^{\prime \prime }\right)$$

where $a_{1},a_{2},a_{3}$ and $a_{4}$ are unknown to be determined. For finding the values of unknown parameters $a_{1},a_{2},a_{3}$ and $a_{4}$ Taylor series expansions for $y_{i}$ are considered. Expanding $y_{i}$ using the Taylor series and substituting Eq. (2) into Eq. (3) leads to

(4) $$y_{i-1}+hy_{i-1}^{\prime }+\frac{h^{2}}{2}y_{i-1}^{\prime \prime }+\frac{h^{3}}{6}y_{i-1}^{\prime \prime \prime }+\frac{h^{4}}{24}y_{i-1}^{iv}+\ldots =y_{i+1}+h\left[ay_{i-1}^{\prime }+ahy_{i-1}^{\prime \prime }+ah^{2}y_{i-1}^{\prime \prime \prime }+by_{i-1}^{\prime }\right]+h^{2}\left[cy_{i-1}^{\prime \prime }+chy_{i-1}^{\prime \prime \prime }+ch^{2}y_{i-1}^{iv}+dy_{i-1}^{\prime \prime }\right]$$

Collecting the coefficients of $hy_{i-1}^{\prime },h^{2}y_{i-1}^{\prime \prime },h^{3}y_{i-1}^{\prime \prime \prime }$ and $h^{4}y_{i-1}^{iv}$ on both sides of Eq. (4) results in:

(5) $$\begin{array}{c} 1=a_{1}+a_{2}\\ \frac{1}{2}=a_{1}+a_{3}+a_{4}\\ \frac{1}{6}=a_{1}+a_{3}\\ \frac{1}{24}=a_{3} \end{array}\}$$

Solving a set of Eq. (5) gives values of unknown parameters as:

$$a_{1}=\frac{1}{8},a_{2}=\frac{7}{8},a_{3}=\frac{1}{24},a_{4}=\frac{1}{3}$$

Thus, the discretization of Eq. (1) using the proposed scheme is expressed in difference Eqs. (1), (3), and values of unknown parameters can be chosen from (5). Thus, the discretization of Eq. (1) is given as:

$${\bar{y}}_{i}=y_{i-1}+hf_{i-1}+h^{2}f_{y,i-1}$$

$$y_{i}=y_{i-1}+h\left\{\frac{1}{8}{\bar{f}}_{i}+\frac{7}{8}f_{i-1}\right\}+h^{2}\left\{\frac{1}{24}{\bar{f}}_{y.i-1}+\frac{1}{3}f_{y.i-1}\right\}$$

where $f_{y}=\frac{df}{dy}$.

## 3 Stability analysis

This section concerns the stability region of the proposed scheme for the linear differential equation. To do so, consider the linear differential equation,

(6) $$y^{\prime }=\lambda y$$

The first/predictor stage of the proposed numerical scheme for Eq. (6) is given by

$${\bar{y}}_{i}=y_{i-1}+hy_{i-1}^{\prime }+h^{2}y_{i-1}^{\prime \prime }=\left(1+h\lambda +h^{2}\lambda^{2}\right)y_{i-1}$$

The second stage or corrector stage of the proposed scheme for Eq. (6) is expressed as:

$$y_{i}=y_{i-1}+h\left[\frac{1}{8}{\bar{y}}_{i}^{\prime }+\frac{7}{8}y_{i-1}^{\prime }\right]+h^{2}\left[\frac{1}{24}{\bar{y}}_{i}^{\prime \prime }+\frac{1}{3}y_{i-1}^{\prime \prime }\right]$$

(7) $$y_{i}=y_{i-1}+\frac{h}{8}\left[\lambda \left(1+h\lambda +h^{2}\lambda^{2}\right)y_{i-1}+7\lambda y_{i-1}\right]+\frac{h^{2}}{24}\left[\lambda^{2}\left(1+h\lambda +h^{2}\lambda^{2}\right)y_{i-1}+8\lambda^{2}y_{i-1}\right]$$

$$y_{i}=\left[1+\frac{z}{8}\left(1+z+z^{2}\right)+\frac{7}{8}z+\frac{z^{2}}{24}\left(1+z+z^{2}\right)+\frac{1}{3}z^{2}\right]y_{i-1}$$

where z=λh.

**Fig. 1:**
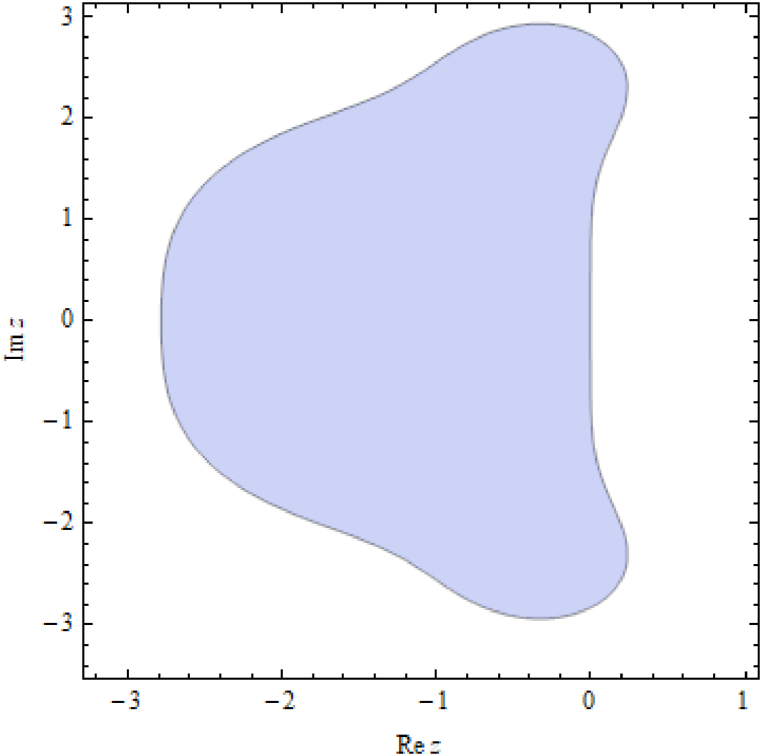
Stability region of the proposed scheme.

The stability condition for Eq. (6) using the proposed scheme can be expressed as,

$$\left|1+z+\frac{z^{2}}{2}+\frac{z^{3}}{6}+\frac{z^{4}}{24}\right|<1\text{,}$$

which is sketched in Fig. 1.

For verifying the consistency of the proposed scheme, consider Eq. (7) with Taylor series expansion for $y_{i}$ as

(8) $$y_{i-1}+hy_{i-1}^{\prime }+O\left(h^{2}\right)=y_{i-1}+h\lambda y_{i-1}+h\left[\frac{\lambda }{8}\left(h\lambda +h^{2}\lambda^{2}\right)+\frac{h}{24}\lambda^{2}\left(1+\lambda h+h^{2}\lambda^{2}\right)+\frac{h\lambda^{2}}{3}\right]y_{i-1}$$

Applying the limit h→0 to Eq. (8), it leads to

$$y_{i-1}^{\prime }=\lambda y_{i}$$

Original Eq. (6) evaluated at grid point ″i−1″. Therefore, the proposed scheme is consistent.

## 4 Problem Formulation

Consider the flow of a steady, incompressible, laminar, non-Newtonian, micropolar fluid across a moving sheet. Allow a velocity to be applied to the sheet. The -axis is perpendicular to the sheet and is positioned along the flow or sheet. The sheet moves abruptly toward the positive x-axis, creating the flow. The governing equations of the flow in the presence of an electric body force can be formulated using [41,42,43,44] and the assumption(s) of boundary layer theory.

(9) $$\frac{\partial u}{\partial x}+\frac{\partial v}{\partial y}=0$$

(10) $$u\frac{\partial u}{\partial x}+v\frac{\partial u}{\partial y}=\nu \frac{\partial^{2}u}{\partial y^{2}}+K_{1}\frac{\partial \sigma_{1}}{\partial y}+\frac{\sigma }{\rho }E_{L}$$

(11) $$\bar{G}\frac{\partial^{2}\sigma_{1}}{\partial y^{2}}-2\sigma_{1}-\frac{\partial u}{\partial y}=0$$

(12) $$u\frac{\partial T}{\partial x}+v\frac{\partial T}{\partial y}=\alpha \frac{\partial^{2}T}{\partial y^{2}}-\frac{1}{\rho c_{p}}\frac{\partial q_{r}}{\partial y}+\frac{b_{1}\sigma }{\rho c_{p}}E_{L}^{2}-\frac{\sigma u}{\rho c_{p}}E_{L}$$

(13) $$u\frac{\partial C}{\partial x}+v\frac{\partial C}{\partial y}=D\frac{\partial^{2}C}{\partial y^{2}}-k_{1}\left(C-C_{\infty }\right)$$

Subject to the boundary conditions

(14) $$\begin{array}{c} u=U_{w},v=0,\sigma_{1}=0,T=T_{w},C=C_{w}\;at\;y=0\\ u\to 0,\sigma_{1}\to 0,T\to T_{\infty },C\to C_{\infty }at\;y\to \infty \end{array}\}$$

where $\nu =\frac{\mu +S}{\rho }$ represents the kinematics viscosity of the fluid, S is a constant characteristic of the fluid, $K_{1}=\frac{S}{\rho }$ is coupling constant. Boundary conditions (14) are specified at and away from the plate. The velocity of the plate, fixed temperature, concentration, and fixed zero angular velocity all affect the boundary condition at the plate. The plate's movement causes the flow, and this movement has an impact on the fluid's nearby layers.

The Joule heating effect is taken into account in the energy equation. The pace of volumetric heat generation is what causes this impact. This occurred due to the ion current's passage warming the fluid. The final term in the energy equation (20) depicts an ion's kinetic work on most of the flow. This term's negative sign indicates that some energy is transformed into the kinetic energy of ions rather than all energy being turned to heat. Think about the transformations [39,40] below.

(15) $$\begin{array}{c} \eta =\sqrt{\frac{a}{\nu }}y,u=U_{w}f^{\prime }\left(\eta \right),v=-\sqrt{a\nu }f\left(\eta \right)\\ E_{x}=E_{0}\;\mathrm{LG}\left(\eta \right),\sigma =\sigma_{0}H\left(\eta \right),\sigma_{1}={\left(\frac{a^{3}}{\nu }\right)}^{\frac{1}{2}}\mathrm{Lg}\left(\eta \right)\\ T=T_{e}+\Delta TL^{2}\theta \left(\eta \right),\varphi =\frac{C-C_{\infty }}{C_{w}-C_{\infty }} \end{array}\}$$

where $\Delta T={\left(T_{w}-T_{e}\right)|}_{x=0}$.

Substituting transformations (15) into Eqs. (9), (10), (11), (12), (13) yields the following dimensionless set of ODEs

(16) $$f^{\prime 2}-ff^{\prime \prime }=f^{\prime \prime \prime }+k\left(1-f^{\prime }\right)G+k_{b}g^{\prime }$$

(17) $$G_{1}g^{\prime \prime }-2g-f^{\prime \prime }=0$$

(18) $$2f^{\prime }\theta -f\theta^{\prime }=\frac{1}{P_{r}}\theta^{\prime \prime }+s_{1}\left(1-f^{\prime }\right)G^{2}-s_{2}f^{\prime }\left(1-f^{\prime }\right)G+\frac{4}{3}\frac{R_{d}}{P_{r}}\theta^{\prime \prime }$$

(19) $$-f\varphi^{\prime }=\frac{1}{S_{c}}\varphi^{\prime \prime }-\gamma \varphi$$

subject to the dimensionless boundary conditions

(20) $$\begin{array}{c} f=0,f^{\prime }=1,g=0,\theta =\epsilon ,\varphi =1\;when\;\eta =0\\ f^{\prime }\to 0,g\to 0,\theta \to 0,\varphi \to 0\;when\;\eta \to \infty \end{array}\}$$

where $\varepsilon =\frac{T_{w}-T_{e}}{L^{2}\Delta T}$ is a dimensionless parameter, and also it is assumed in Ref. [38] that $H=1-f^{\prime }$. In Eq. (12), $q_{r}$ is the radiative heat flux, and for this contribution, its linearized form is considered as follows:

$$q_{r}=\frac{-4\sigma^{*}T_{\infty }^{3}}{3k^{*}}\frac{\partial T}{\partial y}$$

where parameters are defined as

$$k_{b}=\frac{K_{1}}{\nu },k=\frac{E_{0}\sigma_{0}}{\rho a^{2}},G_{1}=\frac{\bar{G}a}{\nu },s_{1}=\frac{E_{0}^{2}b_{1}\sigma_{0}}{\rho ac_{p}\Delta T},s_{2}=\frac{E_{0}\sigma_{0}}{\rho c_{p}\Delta T},R_{d}=\frac{4\sigma^{*}T_{e}^{3}}{\bar{k}k^{*}},P_{r}=\frac{\nu }{\alpha },S_{c}=\frac{\nu }{D},\gamma =\frac{k_{1}}{a}$$

The problem is similar since there is no parameter in the dimensionless version of differential equations that contains a variable along a stream-wise coordinate. Solving the problem with one independent variable is possible by downsizing it with two independent variables.

## 5 Solution procedure

The suggested explicit technique is used to solve Eqs. (16), (17), (18), (19), (20). The suggested numerical approach can be used to discretize first-order ODEs because Eqs. (16), (17), (18), (19) are second and third-order ODEs. Therefore, the second and third-order differential equations (16), (17), (18), (19) are reduced into the system of first-order differential equations to overcome this restriction. Some of the boundary conditions stated in Eq. (20) for a reduced system will be regarded as initial conditions, while the remaining initial conditions will be assumed. The collection of differential equations is written as follows:

(21) $$f^{\prime }=f_{1},f\left(0\right)=0$$

(22) $$f_{1}^{\prime }=f_{2},f\left(0\right)=1$$

(23) $$f_{2}^{\prime }=f_{1}^{2}-ff_{2}-k\left(1-f_{1}\right)G+K_{b}g_{1},f_{2}\left(0\right)=x_{1}$$

(24) $$g^{\prime }=g_{1},g\left(0\right)=0$$

(25) $$g_{1}^{\prime }=\frac{1}{G}\;\left(2g+f_{2}\right),g_{1}\left(0\right)=x_{2}$$

(26) $$\theta^{\prime }=\theta_{1},\varphi \left(0\right)=\epsilon$$

(27) $$\theta_{1}^{\prime }=\left(\frac{3P_{r}}{3+4R_{d}}\right)\left(2f_{1}\theta -f\theta_{1}-s_{1}\left(1-f_{1}\right)G^{2}+s_{2}f_{1}\left(1-f_{1}\right)G\right),\theta_{1}\left(0\right)=x_{3}$$

(28) $$\varphi^{\prime }=\varphi_{1},\varphi \left(0\right)=1$$

(29) $$\varphi_{1}^{\prime }=S_{c}\left(-f\varphi_{1}+\gamma \varphi \right),\varphi_{1}\left(0\right)=x_{4}$$

The suggested approach resolves the first-order differential equations given in (21), (22), (23), (24), (25), (26), (27), (28), (29). For the system of the differential equation shown in (21), (22), (23), (24), (25), (26), (27), (28), (29), the first stage, or predictor stage, is represented as follows:

$${\bar{f}}_{i}=f_{i-1}+hf_{1.i-1}+h^{2}f_{2,i-1}$$

$${\bar{f}}_{1,i}=f_{1,i-1}+hf_{2,i-1}+h^{2}\left(f_{1,i-1}^{2}-f_{i-1}f_{2,i-1}-k\left(1-f_{1,i-1}\right)G\left(\eta_{i}\right)-k_{b}g_{1,i-1}\right)$$

$${\bar{f}}_{2,i}=f_{2,i-1}+h\left(f_{1,i-1}^{2}-f_{i-1}f_{2,i-1}-k\left(1-f_{1,i-1}\right)G\left(\eta_{i}\right)-k_{b}g_{1,i-1}\right)+h^{2}\left[f_{1,i-1}f_{2,i-1}-f_{i-1}\left(f_{1,i-1}^{2}-f_{i-1}f_{2,i-1}-k_{b}g_{1,i-1}-k\left(1-f_{1,i-1}\right)G\left(\eta_{i-1}\right)\right)-k\;\left(-f_{2,i-1}G\left(\eta_{i}\right)+\left(1-f_{1,i-1}\right)G^{\prime }\left(\eta_{i}\right)\right)-\frac{k_{b}}{G_{1}}\left(2g_{i-1}+f_{2,i-1}\right)\right]$$

$${\bar{g}}_{i}=g_{i-1}+hg_{1.i-1}+h^{2}g_{2,i-1}$$

$${\bar{g}}_{1,i}=g_{1,i-1}+\frac{h}{G_{1}}\left(2g_{i-1}+f_{2,i-1}\right)+\frac{h^{2}}{G_{1}}\left(2g_{1,i-1}+f_{1,i-1}^{2}-f_{i-1}f_{2,i-1}-k\left(1-f_{1,i-1}\right)G\left(\eta_{i}\right)-k_{b}g_{1,i-1}\right)$$

$${\bar{\theta }}_{i}=\theta_{i-1}+h\theta_{1,i-1}+h^{2}\left(\frac{3P_{r}}{3+4R_{d}}\right)\left(2f_{1,i-1}\theta_{i-1}-f_{i-1}\theta_{1,i-1}-s_{1}\left(1-f_{1,i-1}\right)G^{2}\left(\eta_{i-1}\right)+s_{2}f_{1,i-1}\left(1-f_{1,i-1}\right)G\left(\eta_{i-1}\right)\right)$$

$${\bar{\theta }}_{1,i}=\theta_{1,i-1}+h\left(\frac{3P_{r}}{3+4R_{d}}\right)\left(2f_{1,i-1}\theta_{i-1}-f_{i-1}\theta_{1,i-1}-s_{1}\left(1-f_{1,i-1}\right)G^{2}\left(\eta_{i-1}\right)+s_{2}f_{1,i-1}\left(1-f_{1,i-1}\right)G\left(\eta_{i-1}\right)\right)+h^{2}\left(\frac{3P_{r}}{3+4R_{d}}\right)\left[2f_{2,i-1}\theta_{i-1}+f_{1,i-1}\theta_{1,i-1}-f_{i-1}\left(\frac{3P_{r}}{3+4R_{d}}\right)\left\{2f_{1,i-1}\theta_{i-1}-f_{i-1}\theta_{1,i-1}-s_{1}\left(1-f_{1,i-1}\right)G^{2}\left(\eta_{i-1}\right)+s_{2}f_{1,i-1}\left(1-f_{1,i-1}\right)G\left(\eta_{i-1}\right)\right\}-s_{1}\left\{-f_{2,i-1}G^{2}\left(\eta_{i-1}\right)+2\left(1-f_{1,i-1}\right)G\left(\eta_{i-1}\right)G^{\prime }\left(\eta_{i-1}\right)\right\}+s_{2}\left\{f_{2,i-1}\left(1-f_{1,i-1}\right)G\left(\eta_{i-1}\right)+f_{1,i-1}\left(-f_{2,i-1}\right)G\left(\eta_{i-1}\right)+f_{1,i-1}\left(1-f_{1,i-1}\right)G^{\prime }\left(\eta_{i-1}\right)\right\}\right]$$

$${\bar{\varphi }}_{i}=\varphi_{i-1}+h\varphi_{1,i-1}+h^{2}S_{c}\left(-f_{1,i-1}\varphi_{1,i-1}+\gamma \varphi_{i-1}\right)$$

$${\bar{\varphi }}_{1,i}=\varphi_{1,i-1}+hS_{c}\left(-f_{i-1}\varphi_{1,i-1}+\gamma \varphi_{i-1}\right)+h^{2}S_{c}\left(-f_{1,i-1}\varphi_{1,i-1}-f_{i-1}S_{c}\left(-f_{i-1}\varphi_{1,i-1}+\gamma \varphi_{i-1}\right)+\gamma \varphi_{1,i-1}\right)$$

The second stages of the proposed scheme for discretizing differential equations given in (21), (22), (23), (24), (25), (26), (27), (28), (29) are expressed in the form:

$$f_{i}=f_{i-1}+h\left[\frac{1}{8}{\bar{f}}_{1,i}+\frac{7}{8}\;f_{1,i-1}\right]+h^{2}\left[\frac{1}{24}{\bar{f}}_{2,i}+\frac{1}{3}f_{2,i-1}\right]$$

$$f_{1,i}=f_{1,i-1}+h\left[\frac{1}{8}{\bar{f}}_{2,i}+\frac{7}{8}f_{2,i-1}\right]+h^{2}\left[\frac{1}{24}\left\{{\bar{f}}_{1,i}^{2}-{\bar{f}}_{i}{\bar{f}}_{2,i}-k\left(1-{\bar{f}}_{1,i}\right)G\left(\eta_{i}\right)-k_{b}{\bar{g}}_{1.i}\right\}+\frac{1}{3}\left\{f_{1,i-1}^{2}-f_{i-1}f_{2,i-1}-k\left(1-f_{1,i-1}\right)G\left(\eta_{i-1}\right)-k_{b}g_{1,i-1}\right\}\right]$$

$$f_{2,i}=f_{2,i-1}+h\left[\frac{1}{8}\left\{{\bar{f}}_{1,i}^{2}-{\bar{f}}_{i}{\bar{f}}_{2,i}-k\left(1-{\bar{f}}_{1,i}\right)G\left(\eta_{i}\right)-k_{b}{\bar{g}}_{1,i}\right\}+\frac{7}{8}\left\{f_{1,i-1}^{2}-f_{i-1}f_{2.i-1}-k\left(1-f_{1,i-1}\right)G\left(\eta_{i-1}\right)-k_{b}g_{1,i-1}\right\}\right]+h^{2}\left[\frac{1}{24}\left\{{\bar{f}}_{1,i}{\bar{f}}_{2,i}-{\bar{f}}_{i}\left({\bar{f}}_{1,i}^{2}-{\bar{f}}_{i}{\bar{f}}_{2,i}-k_{b}{\bar{g}}_{1,i}-k\left(1-{\bar{f}}_{1,i}\right)G\left(\eta_{i}\right)\right)-k\left(-{\bar{f}}_{2,i}G\left(\eta_{i}\right)+\left(1-{\bar{f}}_{1,i}\right)G^{\prime }\left(\eta_{i}\right)\right)\right\}+\frac{1}{3}\left\{f_{1.i-1}f_{2.i-1}-f_{i-1}\left(f_{1.i-1}^{2}-f_{i-1}f_{2.i-1}-k_{b}g_{1.i-1}-k\left(-f_{1.i-1}\right)G\left(\eta_{i-1}\right)\right)-k\left(-f_{2.i-1}G\left(\eta_{i-1}\right)+\left(1-f_{1.i-1}\right)G^{\prime }\left(\eta_{i-1}\right)\right)\right\}\right]$$

$$g_{i}=g_{i-1}+h\left[\frac{1}{8}{\bar{g}}_{1,i}+\frac{7}{8}g_{1,i-1}\right]+\frac{h^{2}}{G_{1}}\left[\frac{1}{24}\left\{2{\bar{g}}_{i}+{\bar{f}}_{2,i}\right\}+\frac{1}{3}\left\{2g_{i-1}+f_{2,i-1}\right\}\right]$$

$$g_{1,i}=g_{1,i-1}+\frac{h}{G_{1}}\left[\begin{array}{c} \frac{1}{8}\left\{2{\bar{g}}_{i}+{\bar{f}}_{2,i}\right\}+\\ \frac{7}{8}\left\{2g_{i-1}+f_{2,i-1}\right\} \end{array}\right]+\frac{h^{2}}{G_{1}}\left[\begin{array}{c} \frac{1}{24}\left\{\begin{array}{c} {\bar{f}}_{1,i}^{2}-{\bar{f}}_{i}{\bar{f}}_{2,i}-\\ k\left(1-{\bar{f}}_{1,i}\right)G\left(\eta_{i}\right)-k_{b}{\bar{g}}_{1.i} \end{array}\right\}+\\ \frac{1}{3}\left\{\begin{array}{c} f_{1,i-1}^{2}-f_{i-1}f_{2,i-1}-\\ k\left(1-f_{1,i-1}\right)G\left(\eta_{i-1}\right)-k_{b}g_{1,i-1} \end{array}\right\} \end{array}\right]$$

$$\theta_{i}=\theta_{i-1}+h\left[\frac{1}{8}{\bar{\theta }}_{1,i}+\frac{7}{8}\theta_{1,i-1}\right]+h^{2}\left[\frac{P_{r}}{8\left(3+4R_{d}\right)}\left\{2{\bar{f}}_{1.i}{\bar{\theta }}_{i}-{\bar{f}}_{i}\theta_{1.i}-s_{1}\left(1-{\bar{f}}_{1.i}\right)G^{2}\left(\eta_{i}\right)+s_{2}{\bar{f}}_{1.i}\left(1-{\bar{f}}_{1.i}\right)G\left(\eta_{i}\right)\right\}+\frac{P_{r}}{3+4R_{d}}\left\{2f_{1,i-1}\theta_{i-1}-f_{i-1}\theta_{1,i-1}-s_{1}\left(1-f_{1,i-1}\right)G^{2}\left(\eta_{i-1}\right)+s_{2}f_{1,i-1}\left(1-f_{1,i-1}\right)G\left(\eta_{i-1}\right)\right\}\right]$$

$$\theta_{1.i}=\theta_{1.i-1}+h\left[\frac{3P_{r}}{8\left(3+4R_{d}\right)}\left\{2{\bar{f}}_{1.i}{\bar{\theta }}_{i}-{\bar{f}}_{i}{\bar{\theta }}_{1.i}-s_{1}\left(1-{\bar{f}}_{1.i}\right)G^{2}\left(\eta_{i}\right)+s_{2}{\bar{f}}_{1,i}\left(1-{\bar{f}}_{1,i}\right)G\left(\eta_{i}\right)\right\}+\frac{21P_{r}}{8\left(3+4R_{d}\right)}\left\{2f_{1.i-1}\theta_{i-1}-f_{i-1}\theta_{1.i-1}-s_{1}\left(1-f_{1.i-1}\right)G^{2}\left(\eta_{i-1}\right)+s_{2}f_{1.i-1}\left(1-f_{1.i-1}\right)G\left(\eta_{i-1}\right)\right\}\right]+h^{2}\left[\frac{3P_{r}}{24\left(3+4R_{d}\right)}\left\{{2\bar{f}}_{2.i}{\bar{\theta }}_{i}+{\bar{f}}_{1.i}{\bar{\theta }}_{1,i}-{\bar{f}}_{i}\frac{3P_{r}}{3+4R_{d}}\left(2{\bar{f}}_{1.i}{\bar{\theta }}_{i}-{\bar{f}}_{i}{\bar{\theta }}_{1,i}-s_{1}\left(1-{\bar{f}}_{1.i}\right)G^{2}\left(\eta_{i}\right)+s_{2}{\bar{f}}_{1.i}\left(1-{\bar{f}}_{1.i}\right)G\left(\eta_{i}\right)\right)-s_{1}\left(-{\bar{f}}_{2.i}G^{2}\left(\eta_{i}\right)+2\left(1-{\bar{f}}_{1.i}\right)G\left(\eta_{i}\right)G^{\prime }\left(\eta_{i}\right)\right)+s_{2}\left({\bar{f}}_{2.i}\left(1-{\bar{f}}_{1.i}\right)G\left(\eta_{i}\right)+{\bar{f}}_{1.i}\left(-{\bar{f}}_{2.i}\right)G\left(\eta_{i}\right)+{\bar{f}}_{1.i}\left(1-{\bar{f}}_{1.i}\right)G^{\prime }\left(\eta_{i}\right)\right)\right\}+\frac{P_{r}}{3+4R_{d}}\left\{2f_{2,i-1}\theta_{i-1}+2f_{1,i-1}\theta_{1,i-1}-f_{1,i-1}\theta_{1,i-1}-f_{i-1}\left(\frac{3P_{r}}{3+4R_{d}}\right)\left(2f_{1,i-1}\theta_{i-1}-f_{i-1}\theta_{1,i-1}-s_{1}\left(1-f_{1,i-1}\right)G^{2}\left(\eta_{i-1}\right)+s_{2}f_{1,i-1}\left(1-f_{1,i-1}\right)G\left(\eta_{i-1}\right)\right)-s_{1}\left(-f_{2,i-1}G^{2}\left(\eta_{i-1}\right)+2\left(1-f_{1,i-1}\right)G\left(\eta_{i-1}\right)G^{\prime }\left(\eta_{i-1}\right)\right)+s_{2}\left(f_{2,i-1}\left(1-f_{1,i-1}\right)G\left(\eta_{i-1}\right)-f_{1,i-1}f_{2,i-1}G\left(\eta_{i-1}\right)+f_{1,i-1}\left(1-f_{1,i-1}\right)G^{\prime }\left(\eta_{i-1}\right)\right)\right\}\right]$$

$$\varphi_{i}=\varphi_{i-1}+h\left[\frac{1}{8}{\bar{\varphi }}_{1,i}+\frac{7}{8}\varphi_{1,i-1}\right]+h^{2}\left[\frac{S_{c}}{24}\left\{-{\bar{f}}_{i}{\bar{\varphi }}_{1.i}+\gamma {\bar{\varphi }}_{i}\right\}+\frac{S_{c}}{3}\left\{-f_{i-1}\varphi_{1,i-1}+\gamma \varphi_{i-1}\right\}\right]$$

$$\varphi_{1,i}=\varphi_{1,i-1}+h\left[\frac{S_{c}}{8}\left\{-{\bar{f}}_{i}{\bar{\varphi }}_{1,i-1}+\gamma {\bar{\varphi }}_{i}\right\}+\frac{7S_{c}}{8}\left\{-f_{i-1}\varphi_{1,i-1}+\gamma \varphi_{i-1}\right\}\right]+h^{2}\left[\frac{S_{c}}{24}\left\{{-\bar{f}}_{1,i}{\bar{\varphi }}_{1.i}-S_{c}{\bar{f}}_{i}\left(-{\bar{f}}_{i}{\bar{\varphi }}_{1.i}+\gamma {\bar{\varphi }}_{i}\right)+\gamma {\bar{\varphi }}_{1.i}\right\}+\frac{S_{c}}{3}\left\{-f_{1,i-1}\varphi_{1,i-1}-S_{c}f_{i-1}\left(-f_{i-1}\varphi_{1,i-1}+\gamma \varphi_{i-1}\right)+\gamma \varphi_{1,i-1}\right\}\right]$$

## 6 Results and discussions

This contribution offers an explicit method for solving both linear and nonlinear ODEs. Each first-order differential equation in the system must have its first and second-order derivatives of the dependent variable evaluated for the scheme to be conditionally stable. It offers accuracy of the fourth order. Unlike the traditional fourth-order Runge-Kutta approach, the suggested system uses second-order derivatives and is built in fewer steps. The approach is conditionally convergent because it is more accurate than first-order. The requirement for stability is the requirement for the scheme to converge. The stability condition must be met for convergence if the step size is set this way. The shooting approach is the method for handling differential equations that are provided. The proposed approach serves as the foundation for the shooting method, and the Matlab solver fsolve is used to identify equation solutions. For uncertain initial conditions, the Matlab solver needs just one set of educated assumptions before beginning an iterative process to find a solution. The shooting-based technique finds the solution using guessed beginning conditions and the residuals of provided boundary conditions for the first guess specified for fsolve. When residuals are discovered, fsolve attempts to find a new initial guess for the unknowns after giving another initial guess. The residuals are subjected to this process until they approach zero. Since the proposed scheme is explicit, an iterative procedure will not be required to solve differential equations using given and assumed initial conditions. The main advantage of using explicit schemes in shooting methods is to choose only one set of initial guesses for the Matlab solver fsolve. Otherwise, the initial guess for the Matlab solver must be updated when the implicit scheme is employed for solving differential equations in some cases. Therefore, by considering explicit shooting method schemes, computation time can be decreased. Theoretically, the proposed scheme's order of accuracy is four because it is built using Taylor series expansions. The freedom to employ differential equations without linearizing them is another benefit of using explicit schemes. Therefore, it is unnecessary to linearize the set of nonlinear differential equations before solving them, which may have been the case with implicit systems.

**Fig. 2:**
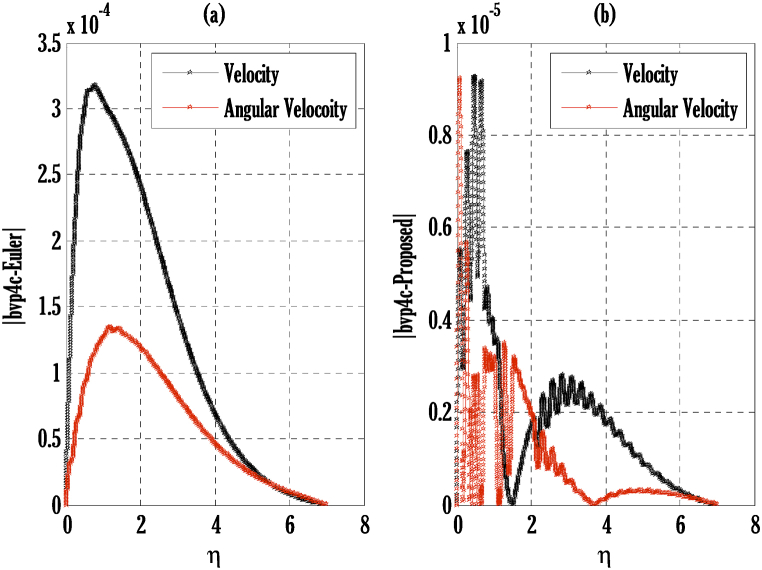
Comparison of the proposed scheme (b) with the existing scheme (a) for velocity and angular velocity profiles using $G_{1}=1,k=0.4,k_{b}=0.1,P_{r}=1.7,R_{d}=0.2,s_{1}=0.1,s_{2}=0.1,\varepsilon =1,S_{c}=1.5,\gamma =0.9,N=3000$.

**Fig. 3:**
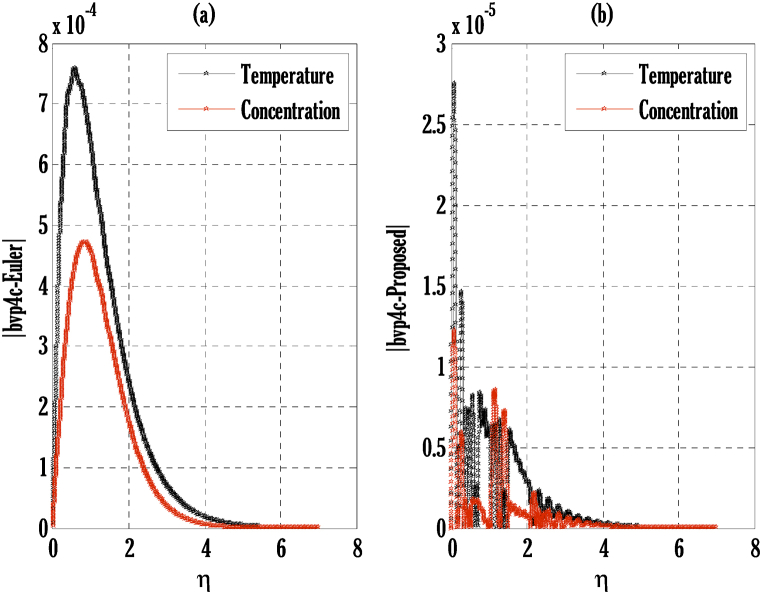
Comparison of the proposed scheme (b) with the existing scheme (a) for temperature and concentration profiles using $G_{1}=1,k=0.4,k_{b}=0.1,P_{r}=1.7,R_{d}=0.2,s_{1}=0.1,s_{2}=0.1,\varepsilon =1,S_{c}=1.5,\gamma =0.9,N=3000$.

**Fig. 4:**
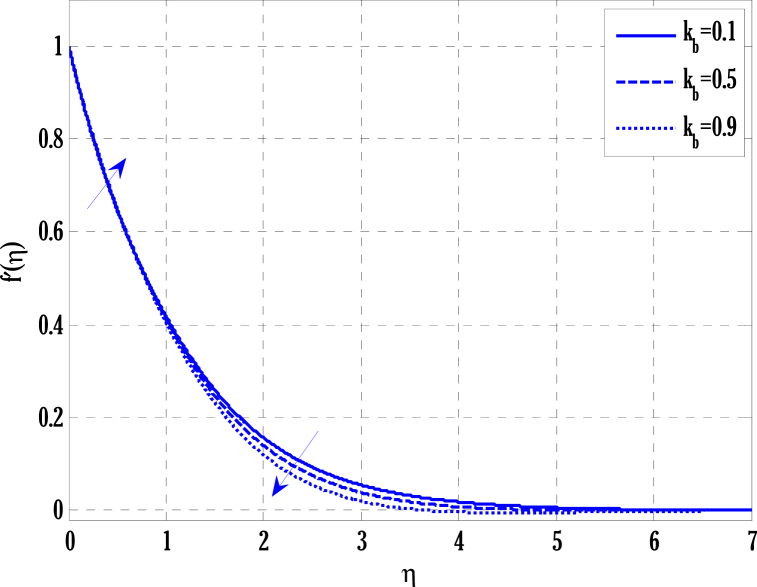
Variation of coupling constant parameter on velocity parameter using $G_{1}=1.7,k=0.5,G\left(\eta \right)=1/\left(1+\eta^{4}\right),N=2000$.

**Fig. 5:**
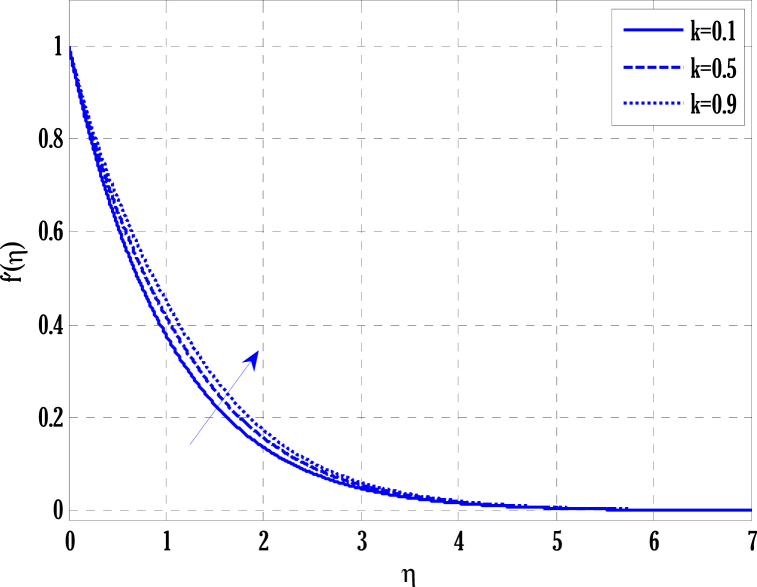
Variation of electro hydrodynamics number on velocity profile using $G_{1}=1.7,k_{b}=0.1,G\left(\eta \right)=1/\left(1+\eta^{4}\right),N=2000$.

**Fig. 6:**
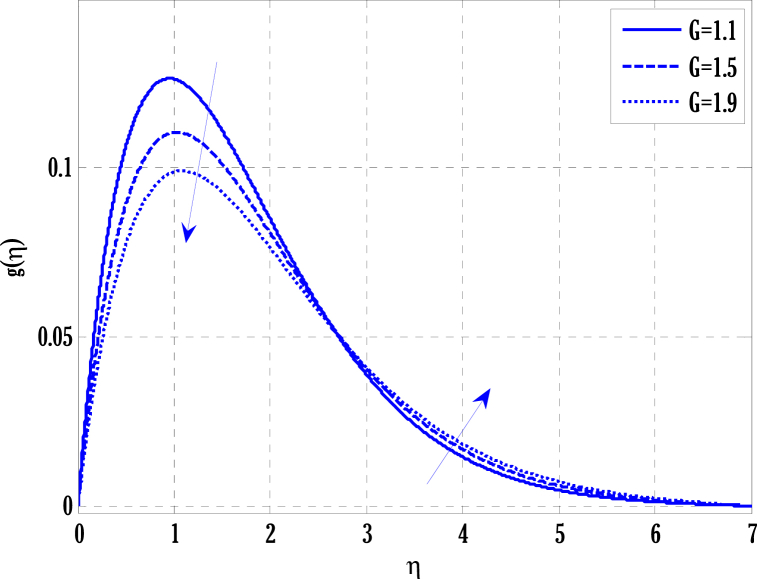
Variation of microrotation parameter on angular velocity using $k=0.5,k_{b}=0.5,G\left(\eta \right)=1/\left(1+\eta^{4}\right),N=2000$.

**Fig. 7:**
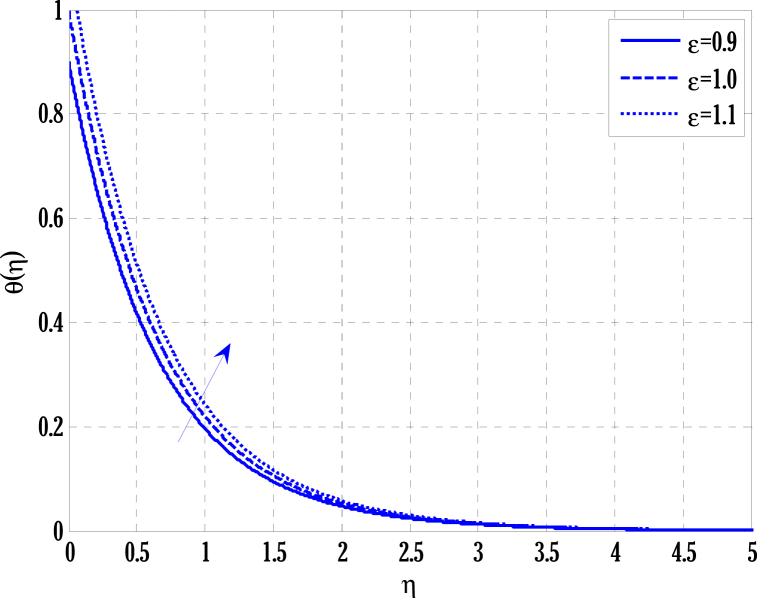
Variation of parameter ε on temperature profile using $G_{1}=1.5,k=0.5,k_{b}=0.1,P_{r}=2,R_{d}=0.5,s_{1}=0.1,s_{2}=0.3,G\left(\eta \right)=1/\left(1+\eta^{4}\right),N=2000$.

**Fig. 8:**
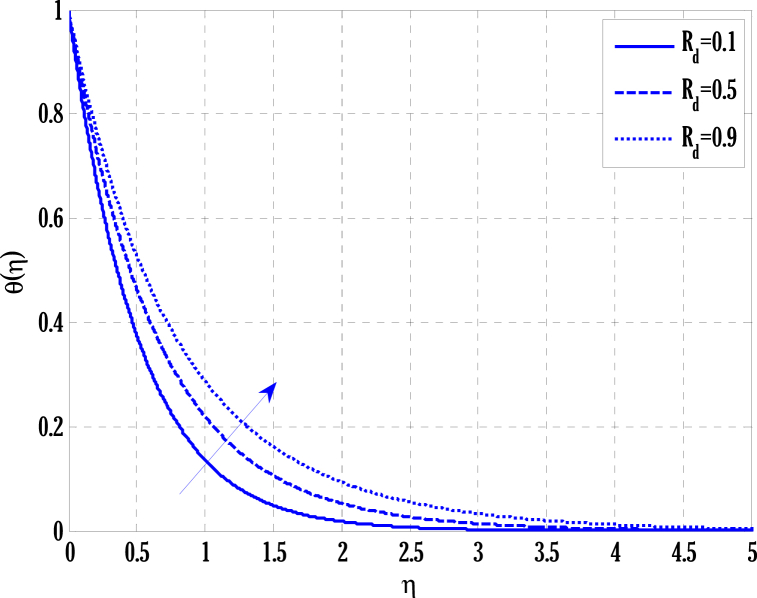
Variation of radiation parameter on temperature profile using $G_{1}=1.5,k=0.5,k_{b}=0.1,P_{r}=2,\varepsilon =1,s_{1}=0.1,s_{2}=0.3,G\left(\eta \right)=1/\left(1+\eta^{4}\right),N=2000$.

**Fig. 9:**
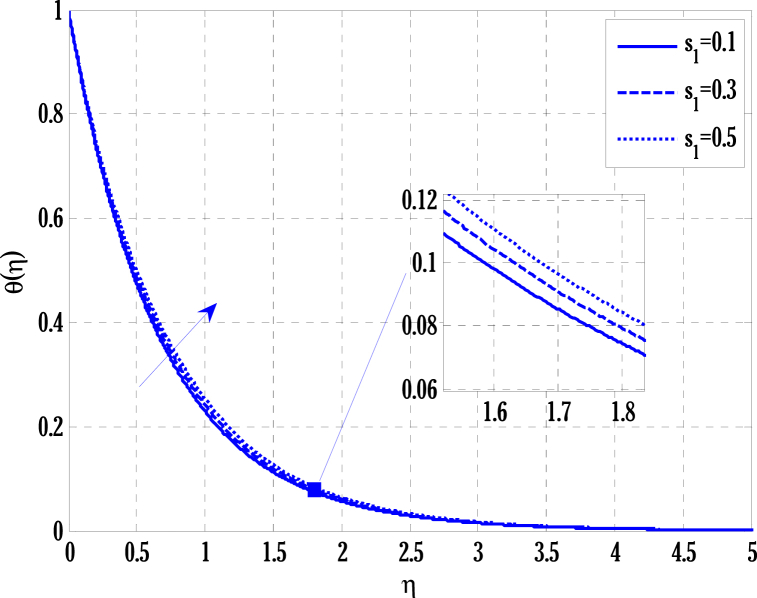
Variation of Joule heating parameter on temperature profile using $G_{1}=1.5,k=0.5,k_{b}=0.1,P_{r}=2,R_{d}=0.5,s_{2}=0.1,G\left(\eta \right)=1/\left(1+\eta^{4}\right),N=2000$.

**Fig. 10:**
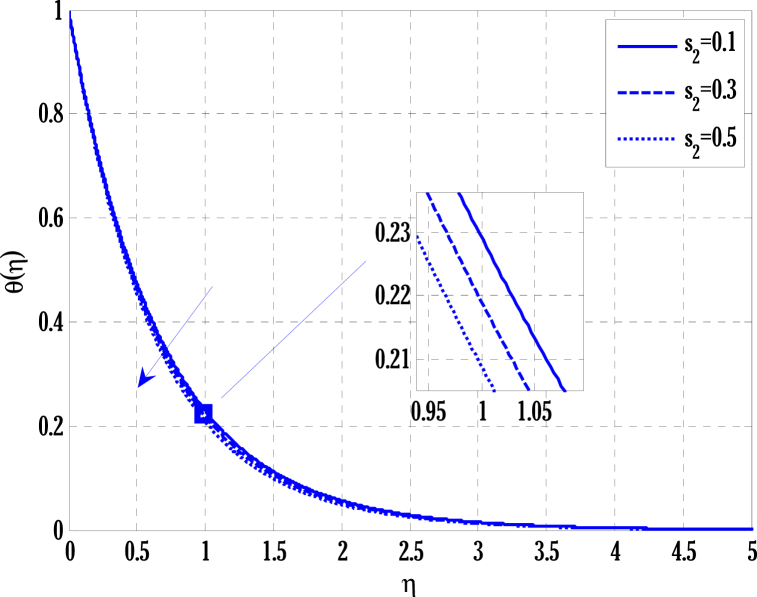
Variation of ion kinetic work parameter on temperature profile using $G_{1}=1.5,k=0.5,k_{b}=0.1,P_{r}=2,R_{d}=0.5,s_{1}=0.1,\varepsilon =1,G\left(\eta \right)=1/\left(1+\eta^{4}\right),N=2000$.

**Fig. 11:**
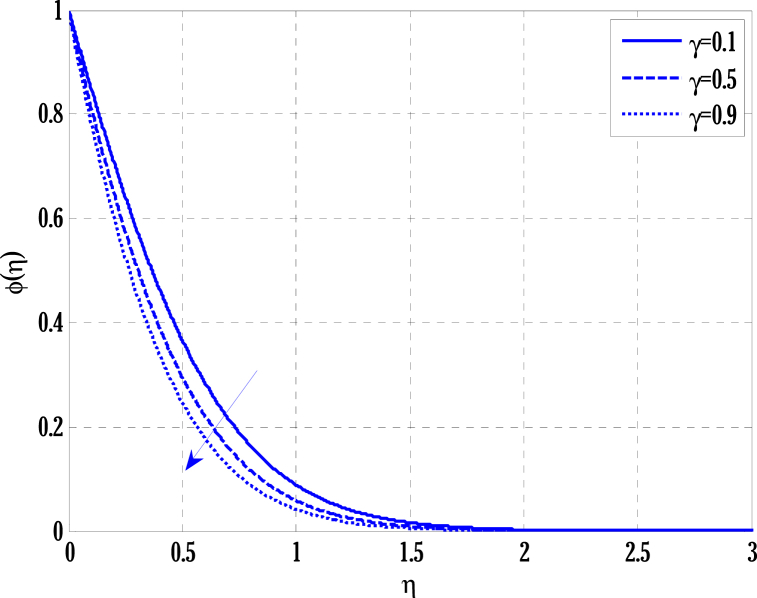
Variation of reaction rate parameter on concentration profile using $G_{1}=1.5,k=0.5,k_{b}=0.1,S_{c}=4,G\left(\eta \right)=1/\left(1+\eta^{4}\right),N=2000$.

Fig. 2, Fig. 3 for the numerical experiment show the proposed scheme's solution with higher accuracy. The first-order Euler and the explicit and conditionally stable proposed techniques are used to compare the solutions obtained for system (16)–(20) because it does not have an exact solution; a numerical solution produced by a Matlab solver is used. Another Matlab solver is used to solve the differential equations (16), (17), (18), (19), (20). Differential equations can be solved using this Matlab solver, which offers fourth- or fifth-order precision. The absolute inaccuracy for each of the four studied profiles—velocity, angular velocity, temperature, and concentration is displayed in Fig. 2, Fig. 3. The absolute difference between the proposed/Euler techniques and the Matlab solver determines the magnitude of the error. Compared to the first-order forward Euler technique, the absolute error produced using the proposed scheme is lower. Fig. 4 considers the effect of the coupling constant parameter on the velocity profile. The velocity profile exhibits a dual behaviour when the coupling constant parameters increase in value. Near the plate, the velocity profile shows a modest increase. However, distant from the plate, the velocity profile de-escalates. Increasing the coupling constant parameter makes it possible to interpret the decay in the velocity profile as a reduction in kinematic viscosity. As a result, the fluid flow's resistance rises, which causes the velocity profile to flatten. Fig. 5 illustrates how the electrohydrodynamics number affects the velocity profile. The velocity profile escalates by growing values of the hydrodynamics number. This happened due to an increase in the strength of electric body forces by growing values of the electrohydrodynamics number, leading to growth in the velocity profile. The influence of the micro rotational parameters on the angular velocity is depicted in Fig. 6. Growing values of the micro rotation parameter in Fig. 6 show the dual behaviour of angular velocity. Fig. 7 illustrates how a parameter affects the temperature profile. As parameter values rise, the temperature profile gets warmer. The increase in the temperature difference between the wall and external fluid temperatures causes the temperature profile to increase. Fig. 8 illustrates the impact of changing radiation factors on the temperature profile. As the radiation parameter is increased, the temperature profile increases. This change in temperature profile results from the fluid's ability to hold more heat due to radiation entering it as the radiation parameter rises. Fig. 9 illustrates how the Joule heating parameter affects the temperature profile. The temperature profile can be observed to increase with increasing values of the Joule heating parameter. As the Joule heating parameter grows, the fluid that transfers energy from the applied electric potential is heated by more Joules. The ion kinetic work parameter's relationship to the temperature profile is examined in Fig. 10. Increasing the values of the ion kinetic work parameter degrades. The conversion of energy to the kinetic energy of ions increases when the ion kinetic work parameter rises because not all energy is lost to heat. Still, a portion is converted to the kinetic energy of ions. This causes the temperature profile to fall since the amount of energy converted to the kinetic energy of ions increases. Fig. 11 depicts the impact of the reaction rate parameter on the concentration profile. Rising values of the reaction rate parameter in Fig. 11 show the deterioration in the concentration profile. The fluid's impurity growth due to the increase in the reaction rate parameter causes the concentration profile to degrade. Any numerical value for a dimensionless parameter in Eqs. (16), (17), (18), (19) may be considered, subject to the convergence of a Matlab program with the proper step size. The physical phenomenon underlying the flow problem may affect the numerical values of dimensionless parameters.

To validate the presented results, a comparison is made for the results obtained by the proposed scheme with those given in past research. For making a comparison in Table 1, an extra term $-k_{p}f^{\prime }$ is included in the reduced momentum Eq. (24). The results are displayed in Table 1 by varying values of $k_{p}$. Table 2 shows the numerical values for $-f^{\prime \prime }\left(0\right)$, $-\theta^{\prime }\left(0\right)$ and $-\varphi^{\prime }\left(0\right)$ by varying the electrohydrodynamics number, micro rotation parameter, Joule heating parameter, ion kinetic work parameter, Prandtl number, and Schmidt number. The increase in $-f^{\prime \prime }\left(0\right)$ is found by rising values of micro rotation parameter and $-f^{\prime \prime }\left(0\right)$ decays by rising values of electrohydrodynamics number. The $-\theta^{\prime }\left(0\right)$ It grows by incrementing the ion kinetic work parameter and Prandtl number and decays by raising the Joule heating parameter. The increase in $-\theta^{\prime }\left(0\right)$ due to rise in Prandtl number is the consequence of decay in thermal conductivity because thermal conductivity and thermal diffusivity can be considered as directly proportional to each other, and Prandtl number and thermal diffusivity can be considered inversely proportional to each other. So, by increasing the Prandtl number, thermal conductivity de-escalates, leading to decay in conductive heat transfer, and therefore, the local Nusselt number rises. The numerical values of $-\varphi^{\prime }\left(0\right)$ rise by enhancing the Schmidt number. The rise in local Sherwood number due to the increase in Schmidt number is the consequence of de-escalation in mass diffusivity, leading to a low diffusion rate.

**Table 1:** Comparison of the proposed scheme with past research for finding numerical values of $-f^{\prime \prime }\left(0\right)$ using $k=0,k_{b}=0,N\left(no.\;of\;grid\;points\right)=3000$.

$k_{p}$	Yih [45]	Hayat et al. [46]	Proposed
0.0	1.0000	1.000000	1.000104
0.5	1.2247	1.224747	1.224816
1.0	1.4142	1.414217	1.414222
1.5	1.5811	1.581147	1.581154
2.0	1.7321	1.732057	1.732055

**Table 2:** List of numerical values for $-f^{\prime \prime }\left(0\right),-\theta^{\prime }\left(0\right),-\varphi^{\prime }\left(0\right)$ with varying $k,G_{1},s_{1},s_{2}$$,P_{r}$ and $S_{c}$ using $K_{b}=0.1,R_{d}=0.1,\varepsilon =1,\gamma =0.9,N=3000$.

k	$G_{1}$	$s_{1}$	$s_{2}$	$P_{r}$	$S_{c}$	$-f^{\prime \prime }\left(0\right)$	$-\theta^{\prime }\left(0\right)$	$-\varphi^{\prime }\left(0\right)$
0.1	0.4	0.1	0.3	0.7	1	0.9722	1.0136	1.1334
0.4						0.9166	1.0320	1.1380
	0.7					0.9186	1.0323	1.1381
	1.0					0.9197	1.0325	1.1382
		0.01				0.9197	1.0417	1.1382
		0.7				0.9197	0.9713	1.1382
			0.01			0.9197	0.9487	1.1382
			0.5			0.9197	0.9869	1.1382
				0.9		0.9197	1.1467	1.1382
				1.7		0.9197	1.6614	1.1382
					0.5	0.9197	1.6614	0.7907
					1.5	0.9197	1.6614	1.4080

## 7 Conclusion

On two grid points, an explicit system has been built. The stability of the scheme for linear ODE was also presented, and the method proved fourth-order correct. The proposed strategy used a shooting approach to address the system of ODEs created by applying transformations to the governing equations of the examined flow phenomenon. A comparison has been done to verify the provided results. The important takeaways can be summarized as follows.

• Fourth order, Runge-Kutta and the proposed scheme had the same stability region.
• The results demonstrate that the temperature profile rises as ion kinetic effort and Joule heating parameters increase.
• By increasing electrohydrodynamics number values, the velocity profile increased.
• The temperature profile rose by increasing the Joule heating parameter's values.
• The increase in ion kinetic work parameter values has caused the temperature profile to increase.

The proposal can address problems in various research and engineering fields in addition to existing methods. Following the completion of this work, it will be possible to propose other applications for the currently employed methodology if desired [47,48,49]. The proposed scheme can be further applied to solve different problems in science and engineering with high accuracy.

## Data availability statement

Data included in article/supp. material/referenced in the article.

## Additional information

No additional information is available for this paper.

## CRediT authorship contribution statement

**Yasir Nawaz:** Conceptualization, Formal analysis, Investigation, Methodology. **Muhammad Shoaib Arif:** Conceptualization, Investigation, Methodology, Supervision, Validation, Writing – original draft. **Kamaleldin Abodayeh:** Data curation, Project administration, Resources, Validation, Visualization, Funding acquisition. **Muhammad Usman Ashraf:** Data curation, Formal analysis, Investigation, Resources. **Mehvish Naz:** Resources, Software, Validation, Visualization, Writing – review & editing.

## Declaration of competing interest

The authors declare that they have no known competing financial interests or personal relationships that could have appeared to influence the work reported in this paper.

## Nomenclature with SI units

$u^{*}$: Horizontal components of velocity $\left(m.s^{-1}\right)$
$y^{*}$: Cartesian coordinate (m)
$R_{d}$: Radiation parameter
ρ: Density of fluid $\left(kg.\;m^{-3}\right)$
C: Concentration of fluid $\left(mol.m^{-3}\right)$
ν: Kinematic viscosity $\left(m^{2}.s^{-1}\right)$
$c_{p}$: Sspecific heat capacity $\left(J.{\text{kg}}^{-1}.K^{-1}\right)$
$E_{L}$: Strength of electric field $\left(volt.m^{-1}\right)$
γ: Reaction rate
$k_{1}$: Reaction rate parameter $\left(s^{-1}\right)$
$k_{b}$: coupling constant parameter
k: Electro-hydrodynamic number
$P_{r}$: Prandtl number
$G_{1}$: micro rotation parameter
$s_{1}$: Joule heating parameter
$s_{2}$: ion kinetic work parameter
σ: Electrical conductivity of the fluid $\left(S.m^{-1}\right)$
T: Temperature of fluid (K)
$T_{w}$: Temperature of fluid at the wall (K)
$T_{\infty }$: Ambient temperature of the fluid (K)
$C_{w}$: Concentration on the wall $\left(mol.m^{-3}\right)$
$C_{\infty }$: Ambient concentration $\left(mol.m^{-3}\right)$
D: Thermophoresis coefficient $\left(m^{2}.s^{-1}\right)$
$b_{1}$: ion charge mobility $\left(m^{2}.s^{-1}.{volt}^{-1}\right)$
α: Thermal diffusivity $\left(m^{2}.s^{-1}\right)$
μ: Dynamic viscosity $\left(kg.m^{-1}.s^{-1}\right)$
$k^{*}$: mean absorption coefficient $\left({\text{cm}}^{-1}\right)$
$K_{1}$: coupling constant $\left(m^{2}.s^{-1}\right)$
$\sigma_{1}$: Angular velocity $\left(s^{-1}\right)$
$S_{c}$: Schmidt number
γ: reaction rate parameter
$\sigma^{*}$: Stefan Boltzmann constant $\left(J/s\;m^{2}\;K^{4}\right)$
